# A peek behind the curtain: exploring coaching styles within the implementation and sustainment facilitation (ISF) strategy in the substance abuse treatment to HIV care study

**DOI:** 10.1186/s43058-021-00246-2

**Published:** 2021-12-20

**Authors:** James H. Ford, Aaron M. Gilson, Martha A. Maurer, Kimberly A. Hoffman, Bryan R. Garner

**Affiliations:** 1 School of Pharmacy, Social and Administrative Sciences Division, University of Wisconsin, Madison, WI USA; 2 Oregon Health and Science University, Portland, Oregon USA; 3 Portland State University School of Public Health, Portland, Oregon USA; 4 RTI International, Durham, North Carolina United States

**Keywords:** Implementation & Sustainment Facilitation, Grasha-Reichmann, Coaching styles, SAT2HIV

## Abstract

**Background:**

The Grasha-Riechmann teaching styles, which includes three didactic and two prescriptive styles, have been shown to help enhance learning within educational settings. Although an adaption of the Grasha-Riechmann style classification has enabled *coaching* styles to be identified for use as part of quality improvement (QI) initiatives, research has not examined the styles actually utilized by coaches within a QI initiative or how the styles change overtime when the coach is guiding an organization through change implementation. Interactions between coaches and HIV service organization (HSO) staff participating in a large implementation research experiment called the Substance Abuse Treatment to HIV care (SAT2HIV) Project were evaluated to begin building an evidence base to address this gap in implementation research.

**Methods:**

Implementation & Sustainment Facilitation (ISF) Strategy meetings (*n* = 137) between coaches and HSO staff were recorded and professionally transcribed. Thematic coding classifications were developed from the Grasha-Riechmann framework and applied to a purposively selected sample of transcripts (*n* = 66). Four coders independently coded transcripts using NVivo to facilitate text identification, organization, and retrieval for analysis. Coaching style use and changes across the three ISF phases were explored.

**Results:**

Facilitator and formal authority were the two coaching styles predominately used. Facilitator sub-themes shifted from asking questions and providing support to supporting independent action over time. Coaches’ use of formal authority sub-styles shifted notably across time from setting expectations or ensuring preparation to offering affirmation or feedback about changes that the HSO’s were implementing. The use of the delegator or personal model coaching styles occurred infrequently.

**Conclusions:**

The current research extends implementation research’s understanding of coaching. More specifically, findings indicate it is feasible to use the Grasha-Riechmann framework to qualitatively identify coaching styles utilized in a facilitation-based implementation strategy. More importantly, results provide insights into how different coaching styles were utilized to implement an evidence-based practice. Further research is needed to examine how coaching styles differ by organization, impact implementation fidelity, and influence both implementation outcomes and client outcomes.

**Trial registration:**

ClinicalTrials.gov NCT02495402. Registered on July 6, 2015.

**Supplementary Information:**

The online version contains supplementary material available at 10.1186/s43058-021-00246-2.

Contributions to the literature

- This analysis supports the feasibility of using a teaching style conceptual framework to identify coaching approaches in a quality improvement initiative.
- This qualitative coding approach can be applied to coaching transcripts resulting from any facilitation-based implementation strategy, regardless of whether it was an original study aim.
- The qualitative coding approach can be successfully implemented by experienced coders who do not have an expertise in the Implementation & Sustainment Facilitation strategy.

## Background

Teacher, coach, and facilitator—the contextual situation defines our understanding. A teacher is often the individual who is involved in the education of students [1–5]. While a coach is often associated with sports, coach or coaching in the healthcare system is associated with sharing expertise (e.g., physician coaching on surgical checklist) to improve care [6–9]. The terms coach, implementation advisor, and facilitator are frequently used in implementation science research [10–13]. Coaching represents discrete and effective implementation strategies [14]. It involves scheduled and structured interactions between an individual with specific expertise and a variety of staff members who work within a healthcare setting [15, 16].

Regardless of the contextual situation and at the most basic level, the terms teacher, coach, implementation advisor, or facilitator, refer to an expert, either internal or external to the organization, who share their knowledge and expertise with other individuals (e.g., students, change teams) to achieve a specific outcome (e.g., meeting educational standards, winning a sporting event, improving medical skills, or successfully implementing change). Although different terms are used to refer to the same role or responsibility in implementation research, the activities that characterize that role or responsibility can vary greatly and often are not readily apparent or classifiable, as if concealed by a curtain. However, Walunas and colleagues highlight the importance of looking behind the curtain to better understand the active facilitation ingredients [12]. Better understanding coaching is especially important in the context of organizational change, given its multifaceted nature [8]. Additionally, given coaching often involves tailoring of coaching styles to build staff confidence to support change [17–19], it is important to study if and how coaching styles may change over time. Therefore, efforts to identify the use of coaching styles in a quality improvement collaborative to support the implementation of an evidence-based practice, and how coaching styles evolve over time represent just a few of the active ingredients behind the facilitation curtain.

Implementing evidence-based practices into routine health service practice can be a challenge, especially in the field of substance use disorder (SUD) treatment [20–25]. It is particularly important to improve the implementation of SUD services within HIV care settings given the high rates of SUD in individuals served in those settings [26–28]. The substance abuse treatment to HIV care (SAT2HIV) project tested the supplementation of the staff-focused Addiction Technology Transfer Center (ATTC) strategy with the team-focused Implementation & Sustainment Facilitation (ISF) Strategy within HIV service organizations (HSOs) in the USA [29]. For example, HSOs in the ATTC arm received centralized technical assistance related to motivational interview training, had access to tools and a system to evaluate the effectiveness of the motivational interviewing (MI) session, and received feedback on those sessions. HSOs assigned to the ISF strategy organized implementation teams, received support from an external facilitator, or “coach,” who guided the HSO staff in efforts to implement a motivational interviewing-based brief intervention (MIBI) for SUDs, and utilized interactive problem-solving and support to help the staff identify and achieve the project’s implementation goals. The main findings of the SAT2HIV Project were that the ISF strategy significantly improved the consistency and quality of implementation (i.e., implementation effectiveness), as well as decreased the odds of patients using their primary substance at follow-up [30].

Given the positive impact of the ISF strategy, our project team sought to better understand the extent to which different coaching styles were used as part of delivering the ISF strategy. In the SAT2HIV project, the implementation advisor or coach is defined as *an individual with training and experience in assisting organizations with practice improvement and implementation efforts* [29]. In this study, coaching represented an interaction between the coach and staff who were members of the HSO’s implementation team. Interactions between the ISF strategy coach and HSO implementation teams occurred on a regular basis (e.g., monthly) to support and encourage teams to harness skills and resources for achieving systemic change and improvement [31]. In the ISF strategy, the coach utilized structured presentations and tools, which helped maintain a common framework for interactions between the coach and each HSO’s implementation team [29]. Studies suggest that a coach’s experience, expertise, and style of relaying information and providing support may affect their effectiveness and organizational performance [10, 17, 18, 32–36].

### Analytic framework

Different teaching styles have been shown to enhance learning in traditional educational environments [5, 37, 38]. In implementation science as identified in the ERIC strategies, the process of interactive problem solving through a supportive relationship (facilitation) relies on an implementation advisor (i.e., a coach) who provides ongoing consultation to support the implementation of the innovation [15, 16]. Coaching styles have been identified as being part of a surgical coaching framework [8], and coaching styles are an important factor in coach/coachee relationships [8, 11, 39, 40]. To begin understanding the implications of coaching styles, the analytic framework used for the current analysis reflects the Grasha-Riechmann Teaching Style Inventory (TSI). The TSI codifies styles and qualities of teachers into five teaching styles: Delegator, Expert, Facilitator, Formal Authority, and Personal Model [5, 41–43]. Additional file 1 provides a description of the coaching styles. In a study of 200 SUD treatment centers participating in a process improvement initiative, the Grasha-Reichmann model was successfully adapted and tested to identify the primary coaching styles preferred by coaches using the Quality Improvement Coach Teaching Style Inventory (QICTSI) [44, 45]. For example, all coaches (*n* = 17) utilized the facilitator style as a part of their primary approach to coaching.

Despite these encouraging findings, they are highly subjective given that the coaching/teaching traits were identified via self-administered surveys. In addition, research has not yet identified coaching styles utilized by coaches within a quality improvement (QI) initiative. More specifically, a coach possesses multiple coaching styles which could influence the relationship between the coach and organizational staff. As a result, a greater reliance on one or more specific coaching styles may be more valuable during different implementation phases as experienced in the SAT2HIV project, which, in turn, may influence implementation differently. Given the ISF strategy sessions in the SAT2HIV project were recorded and transcribed, there was a unique opportunity to investigate the feasibility of applying the Grasha-Riechmann style codes to qualitative data. Through a thematic analysis of recorded and transcribed ISF strategy sessions, we sought to identify the characteristics prominent within each of the coaching styles. This helps address an important gap in the extant implementation research literature, as few studies have examined the coaching styles employed as part of facilitation-based implementation strategies.

### Objective

We assessed the extent to which coaching style changed over the course of the project’s three phases (preparation, implementation, and sustainment). We hypothesized that coaching style would change over time from didactic styles (Expert, Formal Authority, and Personal Model) to less prescriptive styles (Delegator, Facilitator) as the sites become more proficient and independent. To achieve this objective, we explored the methodological feasibility of using the QICTSI in a qualitative application.

## Methods

### Setting and context

Thirty-nine HSO’s located in 23 states and the District of Columbia within the United States were randomly assigned to either the ATTC strategy (control condition) or the ATTC + ISF strategy (experimental condition). Both conditions have been described in detail previously [29]. Coaching in the ISF strategy occurred during three phases (preparation, implementation, and sustainment), was provided by experienced coaches, and lasted 6 months (18 months in total for the three phases). Virtual meetings (approximately 30–60 min) between the coach and the HSO’s implementation team occurred monthly and focused on activities to support efforts by the HSO to implement the project’s MIBI. During the early part of the implementation phase, typically the second month of the implementation phase, a 4-h in-person ISF strategy meeting was conducted in lieu of the monthly virtual meeting.

### Data

Data collection was conducted through digital recordings of ISF strategy meetings. To be included in the analysis, a HSO had to have at least one recorded meeting from each of the project’s three phases. While 137 meetings were recorded and transcribed from the 20 HSOs randomly assigned to receive the ISF Strategy, 10 of the HSOs only had transcripts from only one or two of the phases either because no meeting occurred, or the meeting was not recorded. As a result, transcripts (*n* = 29) from these HSOs were excluded from the study. Our final sample included 10 HSOs with 108 transcribed interviews that met the inclusion criteria*.* Recordings of ISF strategy meetings were then purposively selected from one of the three phases to achieve the final sample of transcribed meetings for coding. Participants typically included HSO leadership and the two MIBI staff [46]. All meetings were conducted in English and professionally transcribed.

### Coding and analysis

We utilized a case study thematic analysis (TA) approach [47]. We followed the six-step framework as described by Braun and Clark [48]. A codebook with definitions consisting of 5 themes, which mirrored the five major teaching styles in the Grasha-Reichmann framework, was completed (see Additional File 2). Sub-themes were iteratively developed within the facilitator, formal authority, delegator, and personal model coaching styles. We double-coded 10% of the sample transcripts to conduct an intercoder reliability check to achieve greater than 80% inter-coder agreement [49–52]. The study team used thematic analysis with a mixed deductive and inductive approach at the semantic level to analyze key topics [48]. Coding was independently conducted by four researchers (JF, AG, MM, KH) using NVivo. Our coding method was not a black letter analysis, based on the presence of particular words, but rather it was an analysis of the concepts and processes inherent in statements. To address the study objectives, percentages were calculated from the frequency of coded language to determine changes in coaching styles over the three study phases. Recognizing that using percentages to quantify the frequency of coded language is not a traditional qualitative approach, percentages are only presented in tables and figures to demonstrate temporal variations and are not included in the body of this paper.

The study protocol was reviewed by the Institutional Review Board and deemed exempt. The Standards for Reporting Qualitative Research (SRQR) checklist [53] was utilized for this manuscript (Additional File 3).

## Results

A total of 66 ISF strategy meetings were included in the analysis. The meetings averaged 40 min across the three phases with an average of three persons from an HSO per meeting (Table 1). The number of coded segments by coaching style, as well as the distribution of coaching styles across and within the phases, are shown in Table 1 and with a more detailed distribution in Additional File 4. Coaching styles are presented in the order of the frequency with which they were identified.

**Table 1:** Characteristics of the interview sample

	**Characteristics of Coded Interviews by Phase**			
	Preparation	Implementation	Sustainment	Total
# of Coded Interviews	26	21	19	66
% of Coded Interviews by Phase	39.4%	31.8%	28.8%	100.0%
Average # of Minutes per Call	39.07	42.57	37.23	39.65
	**Number of Coded Segments by Coaching Style**			
	Preparation	Implementation	Sustainment	Total
Formal Authority	786	938	431	2,155
Facilitator	481	587	369	1,437
Expert	320	356	243	919
Delegator	61	60	29	150
Personal Model	21	32	12	65
Total Coded Segments	1669	1973	1084	4726
Average Segments per Interview	82.4	93.4	60.6	

### Coaching style: formal authority

**Fig. 1:**
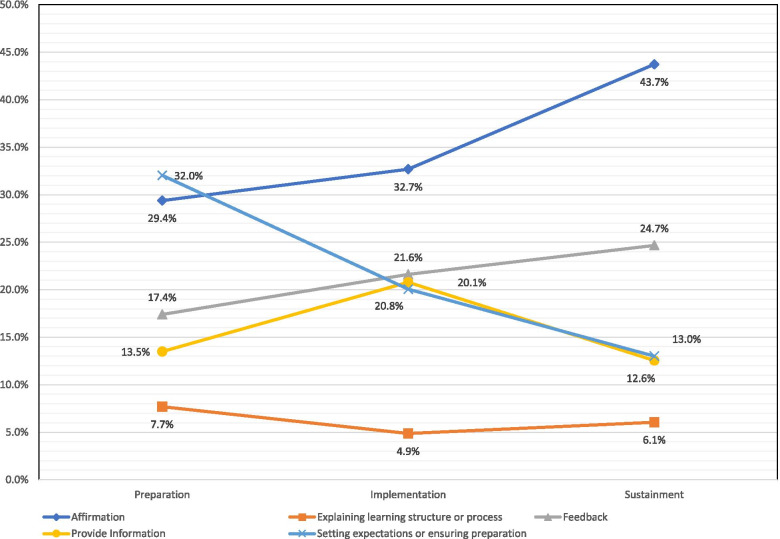
Distribution of formal authority sub-coaching styles by study phase

Cumulative use of statements representative of the formal authority approach showed the greatest frequency of all transcript codes. Formal authority was utilized equally in the preparation and implementation study phases but decreased somewhat during the sustainment phase. However, sub-themes within formal authority varied longitudinally (Fig. 1).

### Affirmation and feedback

Two formal authority sub-themes—affirmation and feedback—occurred most frequently across the different phases (Fig. 1). The coaches’ use of affirmation was often generically in response to statements made by the HSO staff, as illustrated by phrases such as: “that’s great,” “that sounds great,” “thanks, okay, very helpful. I appreciate it,” or “that is a great question.” Feedback occurred when the coaches reacted to the performance by the HSO when integrating the MIBI into their existing workflow or in response to questions from the HSO staff regarding implementation efforts (Table 2). Examples include feedback about plans to implement a “cheat sheet” to guide client interactions (preparation), performance in the consistency of the MI ratings (implementation), or support for decisions not to continue the ISF strategy meetings (sustainment).

**Table 2:** Formal authority coaching sub-theme exemplary quotes

Coaching sub-theme	Exemplary quotes by ISF preparation phase
Feedback	*But what you are describing sounds really healthy to me. That shows that you are engaging and trying to figure out how best to get comfortable … in order to make sure you have the sequence down and that you remember it. (Preparation)* *look at her scores, two different clients, …*. *That shows consistency”. (Implementation)* *… [it’s (referring to the MIBI)] not a tremendous loss and then offered this assurance about the training …. wasn’t a huge component of what got you to this point so far. (Sustainment)*
Providing information	*We’re going to randomly put six of those [clients] into the brief intervention. You’re going to be recording your brief interventions with those people … [and] have monthly calls with ATTC to kind of make sure you are … working on the brief intervention. (Preparation)* *one of the main reasons that we are having them be recorded is so that you all can get feedback on, you know, from an MI grader, about what you did well and areas of improvement. So it’s still an ongoing training and learning experience. (Implementation)* *… putting on the screen the agreement … so we can look at it. I just want to make sure I have the numbers right … $5 per screen, $30 per MI brief intervention, not to exceed $3000 during the time period. (Sustainment)*
Explaining learning structure or process	*If you think about my work … it’s really more on the process side. You know, ATTC is in terms of the content of delivering the training and helping you to have knowledge and information related to the best practice. My job is to support you in terms of the process of taking that knowledge and information and implementing it on the ground, looking at some of the challenges, looking at some of the other issues that may come up, as always come up with any implementation. (Preparation)* *I think it’s very valuable to look at them [feedback reports] and say, okay, which of these items, in terms of the MI-consistent items, did I tend to use more of? Which did I tend to use less of? And how are my adherence ratings on them, competence rating on them? And then the inconsistent behaviors, did I have any of these that I did with any kind of volume? In this case, with this one, there were not any of these. So how do I work on that stuff? (Implementation)* *So doing it yourselves and then being able to share that knowledge with them is probably another step that’s a good one. (Sustainment)*
Setting expectations and ensuring preparation	*You need to take advantage of the in-person training there, when the experts are literally looking over your shoulder doing this, to get the kind of feedback that can set you on the right course. And so moving into that day and taking full advantage of that is key. (Preparation)* *And what we’ll do is be kind of looking at it in the sense of, to what extent were we able to recruit the 12 participants, implement 6 brief interventions, and kind of to what extent was the quality of those brief interventions done, so really kind of 3 things. And we’ll assess them in terms of, you know, there was major room, some room for improvement, and then making any notes about it. (Implementation)* *I would … encourage [you to] really get out and reach and talk to some of the staff that are going to be doing some of these things or perhaps envision to be doing some of these things. See what it’s like for them to hear about it and be asked to do it and be sold on it. Have them test out [the MIBI screening[for a little while and see how effective it is. (Sustainment)*

### Providing information

This sub-theme often involved the coaches providing specific information about the SAT2HIV project and occurred most frequently during the implementation phase as compared to the preparation and sustainment phases. During the preparation and sustainment phases, the coaches often provided information, such as randomly selecting six clients to get the MIBI and then recording those sessions to get feedback from the ATTC staff (Table 2). However, in the implementation phase, a coach explained the purpose behind the MIBI quality ratings by saying “And this isn’t being done to grade you, per se” when affirming to the staff how well they were delivering the MIBI with their clients.

### Explaining learning structure or process

This sub-theme, where the coach described the learning structure or provided information about QI tools and techniques, was one of the least frequently identified for the coaching style. What the coach emphasized within this sub-theme during each study phase varied (Table 2). In the *preparation phase*, coaches provided foundational, high-level, information about the project rationale, and overall goals. Information was also provided about the process with which the implementation team would be involved throughout the project, such as meeting schedules and which implementation team members would participate in various stages, as well as role designations (including the coaches’, as the knowledgeable guiding authority). Indeed, coaches transitioned from providing global information to offering specific descriptions of the process and structure for learning the MIBI and integrating it into practice, including specific tools and mechanisms that the team would use to accomplish continuous QI (e.g., feedback reports and individualized trainings). The *implementation phase* was characterized by the coaches leading the implementation team through activities to think critically about the feedback on the MIBIs and emphasizing the benefits of examining what happened during the MIBIs and how to improve the process by reviewing the manual and/or session recording. In the *sustainment* phase, examples of this sub-theme were the least prevalent because the coaches had already created the foundation and established the learning process and structure. During the sustainment phase, coaches described potential next steps for sustaining the MIBI (e.g., continuing monthly coaching meetings) and guiding discussions about the pros and cons of potential sustainment plans for their HSO given institutional priorities.

### Setting expectations and ensuring preparation

Throughout the study phases, the sub-theme *setting expectations/ensuring preparation* consistently represented a notable proportion of the overall instances of the formal authority coaching style. Sub-theme prevalence decreased across the study phases (Fig. 1). The focus of coaching efforts to set expectations or ensure preparation differed by phase (Table 2). During the preparation phase, often when the coaches were describing the overall purpose of project, they would focus on what was expected from them and the team members to achieve agreed-upon goals. The activities focused on ensuring that team members were prepared (e.g., setting the stage about ATTC and ISF work), addressed implementation climate (i.e., the extent to which implementation was expected, supported, and rewarded), and discussed how each team member will contribute to team preparation. Distinct from long-term high-level expectations, coaches also established short-term expectations—describing meeting agenda items, establishing timeframes and deadlines for certain aspects of the study (e.g., number of participants to recruit and randomize within a certain timeframe), and providing direction about activities the team should accomplish before the next call. This setting of expectations sometimes involved an emotional component, when coaches tried to preemptively alleviate members’ concerns (e.g., acknowledging that a challenge may arise and assuring that it was to be expected) while also motivating members to focus on continuous QI and not be discouraged by challenges. Interestingly, this sub-theme in the implementation phase demonstrated largely similar characteristics as those identified during the preparation phase, except that activities were focused on implementation of the MIBI and preparing for transitioning to the sustainment phase. Compared to the earlier phases, there were the fewest instances of coaches utilizing this sub-theme during the sustainment phase. By this time, the teams had completed most of the meetings and tasks with the coaches, so there was less of a need for coaches to remind teams about upcoming activities of deadlines. Coaches had earlier set expectations about transitioning from implementation to sustainment, emphasizing the continued use of acquired skills/tools to help clients. Later in the sustainment phase, the coaches spent time discussing team expectations for next steps and whether they wanted to sustain the MIBI and continue with the ISF Strategy meetings.

### Coaching style: facilitator

**Fig. 2:**
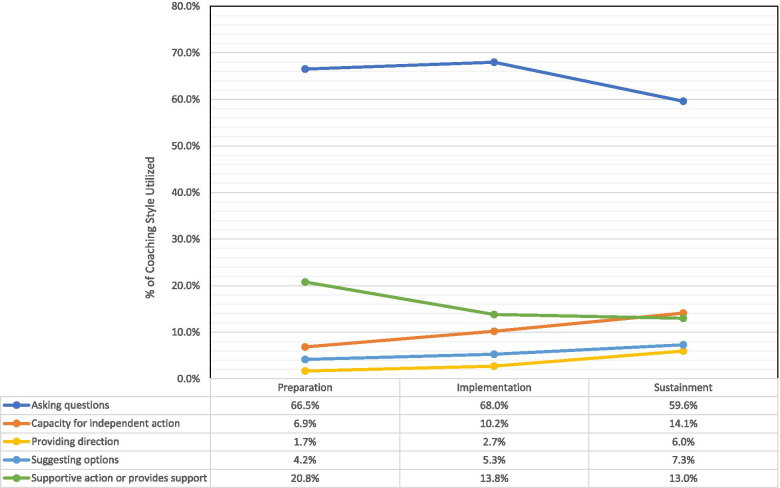
Distribution of facilitator sub-coaching styles by ISF study phase

Of the various coaching styles, the cumulative category of facilitator represented at least a third of all coaching styles over the preparation and implementation periods but fell noticeably during the sustainment phase. It is important to note, however, that this pattern of effect tended to vary somewhat depending on the individual facilitator styles that were applied to the interview transcripts (see Fig. 2 for the longitudinal patterns of effects for each of the components of the facilitator style).

#### Asking questions

Coaches asking questions of team members was the most frequently used sub-theme within the facilitator coaching style and characterized two-thirds of all identified facilitator sub-themes. Although most questions exemplified generic attempts to enquire about the need for additional information or clarification (e.g., “Any questions or concerns right now”), there were several specific instances where the questions were specific to content relevant for each of the 3 study phases (Table 3).

**Table 3:** Facilitator coaching sub-theme exemplary quotes

Facilitator sub-theme	Exemplary quotes by ISF preparation phase
Asking questions	*Any thoughts or questions as you look at this logic model? Does it make sense? Does it fit for you? What do you think?* (Preparation) *So one question to any of you who might have a sense of this, in terms of the screeners, the folks who are additionally brought on to do the part one screening, do you feel like they are confident and comfortable in relation to doing it?* (Implementation) *What do you think might your goals be for this, kind of this sustainability phase? … If we think realistically, what do you think, in terms of trying to maintain some of the work that you have done with this project? … What might our goals be?* (Sustainment)
Capacity for independent action	*As you guys start doing this, you can be thinking about how it’s all going to flow together, and we can make the adjustment to make sure that it will flow together as well as possible.* (Preparation) *I think it [MIBI] could be a useful tool to keep kind of staying dialed into some of these factors that you guys are pointing out and as we are talking about today in terms of good implementation and sustain. But that will be up to you … if you want to do that.* (Implementation) *The idea is, you know, the research has shown that if you have people to continue to do exactly the best practice, they are more likely to sustain it in the longer term. So we are encouraging people to do kind of the whole, complete best practice as we have taught it to you and as you have learned it and done so well with.* (Sustainment)
Providing direction	*Facilitating and enhance focus of the goals on this SAT2HIV project, so what I’m going to be there to do is to kind of keep you focused on those. You know, what are we trying to do? Where are we trying to go with this stuff?* (Preparation) *‘Okay, the guy is out in the waiting room. Let me just take ten minutes and say let me go read the quick summary on pros and cons [of the MIBI] … or the quick summary on developing discrepancies, … just five minutes before I sit down with the person* (Implementation)*.* *And whatever that screening question is [it] is important to fit in there and make sure that it’s appropriate and doable and effective across all the people who are going to be delivering it in the outreach sector and other situations, right* (Sustainment)
Suggesting options	*A client is going to come in for their six-month appointment or maybe their new intake assessment, and [staff] the BI, the part one only staff, is going to screen them and he finds that they are eligible. How is he going to, [as a strategy] how am I going to let [counselor] know that I have this client who wants to participate in the study?* [Preparation] *Maybe …. what would be a good, feasible plan … [is] for you to consciously try to improve for the next couple of BI sessions that you do? Like … whether it be rehearsing it with each other, the group coaching calls, something else, what makes you [comfortable].* (Implementation) *We could kind of explore some of that territory, explore kind of how this is working within your program, as an ongoing process for you to continue to implement what you are doing.* (Sustainment)
Supportive action or provides support	*Here’s what I’m going to do. I’m going to have [research team leader] just shoot [an e-mail], because he’s happy to do this, and it will probably make it easier for you* (Preparation) *I’m trying to do my best to support the process with you so that we can keep the folks flowing, getting the part ones and then the randomized and then doing as many of the part twos as you can* (Implementation) *I’m willing and able and interested in continuing to touch base with you and other team members to talk about how the MI stuff is influencing your work, how you can sustain these skills that you have developed* (Sustainment)

#### Capacity for independent action

Throughout the study phases, coaches took opportunities to develop teams’ capabilities for independent action related to the project. Coaches’ use of this sub-theme also increased substantially throughout the project timeframe (see Fig. 2). As with asking questions, attempts to improve independent activities are characterized by general guidance to achieve this objective, an exemplar of which is “keep trying to do that whenever you can and to give each other pointers.”

However, characteristics of substantive comments tended to vary by study phase (Table 3). The preparation phase dealt with efforts to demonstrate proficiency in the MIBI and the implementation process, with such activities involving brainstorming, training, skill development, resources, communication, planning strategies, and addressing potential challenges. The implementation phase generally consisted of discussions about process and workflow (e.g., recruitment, randomization, delivery of the MIBI) and strategizing in anticipation of the sustainment phase. Finally, the sustainment phase focused on whether and how to continue implementation of the MIBI.

#### Providing direction

Compared to other facilitator coaching styles, coaches provided directions to study team members the least. When examining the specific activities that were captured by this coding, both the preparation and implementation phases involved similar undertakings (Table 3). Such actions included improving the process, staff use of the tools, continued guidance about information needs, training, and protocol adherence, as well as timeline considerations (i.e., anticipating deadlines and goals. These activities largely focused on enhancing implementation plans, including screening and recruitment of client participants, and refining the strategies for working with those client participants. During the sustainment phase, although the same activities were identified as in the previous two phases, additional interests were evident. In the last study phase, coaches provided directions to improve the system and provision of services, transitioning to a sustained activity, assuring fidelity to the service model, and taking a “long-term view” of improving care for clients.

#### Suggesting options

Again, there were few instances of situations where a coach offered suggestions about directions forward on the project. Like facilitator sub-themes associated with capacity and providing direction, the coaches increased use of this sub-theme from the preparation to sustainment phase (Fig. 2). Coaches offered considerations about how implementation team members could work through problems and adjust approaches to achieve appropriate objectives, coordinating meetings before trainings (to assist preparation), and examining strategies that would fit within the HSO culture (Table 3). These recommendations were often based on the coaches’ previous experiences or when compared to ongoing initiatives in other organizations.

#### Supportive action or provides support

Offering to provide support to team members constituted the second-most frequently used facilitator-specific sub-theme. As shown in Fig. 2, the decrease in the use of this sub-theme over the course of the study was much more gradated than for the overall facilitator category. Even given this gradual frequency reduction over time, the types of activities identified as relevant to this coaching style largely remained markedly similar throughout the study phases such as sharing information, including notes of meetings and invitations for next meetings, providing resources (e.g., PowerPoint slides, data collection tools), tasks to accomplish, and experience with other HSOs (Table 3). Additional coach activities involved offering their contact information for quick communication and assistance with the process, helping achieve certain objectives, and addressing problems or questions. Finally, there was a consistent willingness on the part of coaches to offer further training to others who could provide services.

### Coaching style: expert

In the expert coaching style, the coach often possesses knowledge, information, and expertise that is shared to ensure that the organization is ready to implement change and will be successful. Overall, the cumulative use of the Expert coaching style was the third most frequent across the preparation, implementation, and sustainment phases (Table 1). Coaches utilized this style to transmit information about the project, often displaying detailed knowledge about the project or relaying how their experiences might inform the implementation and/or sustainment of the MIBI.

Use of the expert style varied within each phase. In the preparation phase, coaches shared knowledge about planning for organizational change and emphasized that planning alone is not sufficient. Coaches stressed the importance of a comprehensive approach to putting plans into action with a recognition that overcoming current practices would not be easy. For example, they encouraged staff that “the only way to really start to do it is to just practice it, to do the roleplay” and reinforced the message that staff would gain the “knowledge and skill to do [MIBI]” through practice. Coaches also emphasized that proper planning ensures MIBI delivery with consistency and quality by offering specific suggestions such as “staff have to be focused and motivated and have to figure out a way to make sure you don’t disrupt your relationships with the clients.”

Expert coaching styles differed in the implementation and sustainment phases. During implementation, a coach was likely to share a case story or experience. The purpose of the exchange was to promote and encourage engagement by the HSO staff. In other instances, these shared stories re-enforced those actions taken by the HSO staff were consistent with their experiences. For example, one HSO was struggling with continuity after screening a client, especially when they did not show up for the next scheduled appointment. In this case, the coach indicated that he/she had been working with no-shows for the past 10 years and indicated that actions taken by HSO staff was consistent with their experience (Table 5).

In the sustainment phase, the coaches depended more on the expert coaching style as reliance on the formal authority coaching style decreased (Table 1). Specifically, they shared their expertise about how internal challenges (e.g., grant writing) or how the external environment (e.g., regulations) hindered sustainment. More importantly, a coach asked questions, like “What would you want to learn more about brief intervention before you … really committed [MIBI] to the standard operating procedures of the organization?,” to promote efforts to sustain the MIBI intervention. An exchange between the agency and coach highlights the importance of organizational motivation to sustain the change (Table 5).

### Coaching style: delegator

This coaching style was not often utilized by the coaches (Table 1). In this style, the coaches were mostly concerned with developing team capacity to function autonomously by encouraging specific actions or directing the team to seek the support needed to carry out project related activities. *To delegate tasks, t*he coaches also cultivated self-direction, while always ensuring that staff understood they could rely on him/her as a resource, reach out to him/her between meetings if they had any questions, and even offering help with specific tasks (Table 4). With the delegator style, the coaches would offer advice about how to carry out a task and take immediate independent action. Instilling a practice of forethought, so that participants could be thinking of “how you’ll be doing, say, six months from now,” was an important element within the delegator style and enabled participants to better understand what was expected of them. The coach accomplished this by being directive and helping the team understand what was expected or by providing specific directions including what the coach would like the HSO staff to discuss on their coaching calls. The coaches also affirmed independent actions which enabled participants to know they were on the right track with MIBI implementation. In addition to these activities, the coaches would provide guidance about not just what but who might be involved in tasks. For example, the coaches would provide specific direction about who might be on a specific call or involved in a specific project. Finally, delegating tasks to be completed between meetings was commonly observed.

**Table 4:** Delegator coaching sub-theme exemplary quotes

Delegator sub-theme	Exemplary quotes
Being accessible to the team	*“So if there’s ever parts that are feeling difficult, stressful, struggling, questions that you cannot easily get answered, just get with me. Give me a call, send me an email, and then it’s my job to track that down for you and to sort that out for you. That’s how we make sure that you are able to be successful with this work, so I want to invite you at any point to make sure you are doing that.”* *“If there’s any questions, just shoot me an email, give me a call. I mean, you can also speak directly with Brian and/or his team members who are supporting the research process, obviously, in terms of those procedures and protocols. But touch base with me if there’s any questions, or I can talk to them.”* *So if there’s a question, frustration, issue, concern, just shoot me a quick email. I’ll get right on it, get back to you, let you know what’s what. So feel free to do that as we move along.*
Providing specific directions	*“And the timing, it sounds like, is important too in terms of this consensus that you have that, you know, we need a smaller work group that gets rolling, and then we need to work toward including others. And we have to do that, I would say, as soon as possible. But the trick is determining what is as soon as possible?”* *“And maybe what you could do is, as you are doing that, maybe talk to [staff member] about that as well because we are reviewing some of hers. Like, you know, just have her take a look at those and say, okay, [staff member], you can also do the same thing. And you guys could make it into a little, you know, study project between the two of you.”*
Being directive about expectations	*“So each month I’m going to ask you to give me your sense of how it’s going in terms of recruiting people. Is it everything is perfect, some room for improvement, or major room for improvement?”*
Affirming independent actions	*“You’ve been doing some marketing and letting people know that you are doing this. You have people who you are all set to go ahead and recruit and do the part one screen on and then randomize and do the part two. So you have got yourself in good position to get started.”* *“But for the time being, what you are starting to systematize … taking on this role of screening. And that’s why … as you were talking about what it will look like [at HSO] in a couple of months as the study goes away, and, [staff], you were starting to think about how it would just look regularly that everybody’s annual screening has this piece [MIBI] to it where they just regularly get that, that’s very akin to that whole idea.*
Between meeting expectations	*“So between now and the next time we meet, it’s not a big deal, just maybe touch base with each other and think about something you all have implemented as an organization. You can tell me a little bit about that, and we can have a discussion about how you have been doing with that particular implementation and sustainability and then how that’s going to reflect on this project.”* *“There’s one piece of homework that I will have you do between now and the next time we do our call. And in just a minute, we are going to schedule that. I’d like you to think about an implementation that you have done successfully at some point in the past because that’s usually a nice place for us to start talking about implementation. So the first sort of topic for our next call will be to think about those elements of that successful implementation. It will probably reflect some of the strengths of your culture, your team, and we want to harness those.”*

**Table 5:** Exemplary quotes from the expert coaching style

Intervention phase	Exemplary quote
Preparation	*The whole science of organizational change and how institutions really sort of adjust and adapt new ways and new innovations over time to be competitive and be reactive to what clients need and what better treatments and services can be delivered.*
Implementation	*This [project] is contributing to the science that hopefully will be expanded and diffused across the country to help really other … AIDS service organizations pick up what you have sort of learned and made your way through early on in trying out this new service.” I’m a big fan of the gift cards and the whole contingency management, best practice, and using these rewards and incentives, regardless of whether or not you are involved in a research project, because, as you know, they are really effective. They’re nice ways to engage folks and get people’s attention.* *Sounds like you are making some adjustments a little bit further upstream that will help alleviate that for the time being as well. And so, you know, maybe we can hold that off if you choose. Otherwise, I’d be happy to talk about some thoughts or some experiences that I have on that too.*
Sustainment	*You’ve been describing the circumstance and the environment around [agency] is there’s just a, there’s a desire. … But even looking forward, it’s a little bit unclear … on exactly how it may be regenerated in the future on that. But the commitment is still there. And so it would be really neat to be able to come up with a couple of ways to really to drive toward, set as goals, to be able to, [sustain the MIBI]. From my organizational change engineering perspective here, a couple things really stand out, and one is that … you found it very beneficial to your environment, being able to get in and use that motivational interviewing technique to work better with the clients, to really get a, maybe a richer or deeper understanding of some of who they were.*

**Table 6:** Personal model coaching sub-style theme exemplary quotes style

Personal model sub-themes	Exemplary quote
Prior concrete experiences	*“You know, I’ve got a pretty significant background as a clinician in working with a range of different kinds of best practices. And I think that, and I still do some clinical supervision with a number of folks here in my setting.” (Preparation)* *“For example, if I was talking with an organization, and they were saying, oh, and the clients are being such a pain about it, and they keep calling, and they just, they do not even know. We gave them the information, but they are not listening to it, then I would say, oh, this is not going to go well. I’d be leaning over here, even though the jury is not in.” (Preparation)* *“I have found with recordings, if you make it seem like it’s an everyday occurrence, like it’s no big deal, we do this all the time, then it’s a little bit more comfortable for people. And the more you do it, the more it becomes casual for you.” (Implementation)* *“I remember when I was working primarily in direct services and seeing a lot of clients, when I had things going on in my life, either because of bigger systems, issues like the kinds of things we are talking about today or just some major personal stuff happening, you know, those were the moments in time where I was able to sit most comfortably because there was none of that defensiveness. There was just this kind of openness to dealing with whatever difficulties are on the table and just being comfortable with it because that’s the water we are swimming in. So this is okay. We’re just going to be here.” (Sustainment)*
Providing direction without being overly directive	*One thing I would watch is the kind of if there’s almost as many closed-ended as there are open-ended, try and see if you can keep that ratio a little bit farther apart so it’s, there’s a good number more open questions than there are closed.* *Looking at those discrepancies, that’s when I think it’s kind of really useful. I like that one a lot. Is there a way I could maybe kind of take a look at the manual and say, hmm, let me review that one and say maybe those that I’m doing not very much of, maybe I can try next time to do more of those?* *I’m a big fan of the gift cards and the whole contingency management, best practice, and using these rewards and incentives, regardless of whether or not you are involved in a research project, because, as you know, they are really effective. They’re nice ways to engage folks and get people’s attention.*

### Coaching style: personal model

The personal model coaching style was the least utilized style both overall and across the intervention phases. When using the personal model style, the coach teaches by example, often using phrases such as “this is what I would recommend, or this is what I would do” to provide direction without being overly prescriptive (Table 6). The use of prior experiences reflected opportunities for coaches to provide recommendations based on their personal experience or what they might do when faced with a similar situation. An example of this is discussing with the HSO staff about the potential use of gift cards to incentivize clients. When providing direction, language was identified that gently guided HSO staff, providing opinions not just about specific tasks, but approach and style. This was particularly effective where nuance was needed in situations that might have been difficult. For example, when staff were lamenting the amount of paperwork needing to be completed, the coaches offered sympathy. However, the coach, when using the Personal Model, may tend to offer suggestions more frequently to the change team rather than let them explore options and make informed choices about change in their organization.

## Discussion

Coaching is a recommended implementation strategy. Evidence clearly indicates that coaching, in general, is effective and, more specifically, coaching as part of the SAT2HIV project’s ISF strategy improved implementation effectiveness, as well as the effectiveness of the MIBI [30]. This research extends the understanding of coaching in implementation research. It builds on prior research in which the Grasha-Riechmann TSI was adapted to identify coaching styles in QI [45]. We adapted the coaching styles and qualitatively assessed how they were utilized within a QI collaborative to support efforts by HSOs to implement the project’s MIBI for SUDs among people with HIV. The study also examined coaching styles within each study phase and longitudinally.

Coaches rely on their expertise (skills, knowledge, and experience) and personality characteristics (level of respect or trust) to help staff identify and overcome barriers, promote continuous improvement, and facilitate adoption of evidence-based practices [54]. We found that coaches primarily relied on one of three coaching styles—facilitator, formal authority, and expert—as they worked with HSOs overall and within each of the study phases. These results only partially agree with prior research. When interacting with SUD treatment organizations in the NIATx200 initiative, coaches preferred to use either the facilitator, delegator, or personal model as their primary coaching style [45]. The NIATx200 findings were based on a coaches’ self-assessment and in contrast, our analysis based on independent coding of coach transcripts of the actual interaction with the HSOs. These findings suggest that coach perceptions about their coaching styles differ from the styles that they utilized when interacting with staff. This discrepancy may result from the qualitative coding approach being better able to assess and identify the variability in coaching styles that occur both within and throughout the preparation, implementation, and sustainability phases of this study.

Coaching for organizational or individual change is multifaceted. For example, a surgical coaching framework suggests that coaches rely on distinct yet interrelated activities of coaching—setting goals, encouraging, and motivating, and developing and guiding which are supported by coach interpersonal skills including their coaching style [8]. In our study, coaching styles used within each phase (preparation, implementation, and sustainment) shifted both within the phase and over time by coaching style. In these situations, coaches (like other teachers) tailor the learning interchange to individual needs and goals and use active (e.g., a hands-on Plan-Do-Study-Act activity) vs. passive (e.g., a lecture on data collection and measurement) learning approaches to develop skills and build confidence [17–19]. In the context of the SAT2HIV project, coaches changed their styles to guide the HSOs in addressing specific issues within each implementation phase. For example, a greater reliance on the formal authority style (a prescriptive coaching style) during the preparation and implementation phases involved the coach providing feedback about the approach utilized by the HSOs to recruit clients to the stud or to set expectations about what the HSO staff needed to do when implementing the MIBI in their organization. As HSO staff grew more comfortable in their ability to implement change, the coach used less prescriptive coaching styles (e.g., facilitator) to support independent action from staff by suggesting options for how they could implement change. This shift in coaching styles is consistent with the age-old adage of the importance of teaching an individual how to fish. The evolution of the coaching styles as seen in the SAT2HIV Project is consistent with prior research which suggest that facilitation activities evolve over time [11, 55–57].

However, the reliance by the coach on less prescriptive styles (facilitator and delegator) was only partially supported as hypothesized. Our results indicated that the use of less prescriptive styles only changed from the implementation to the sustainment phase but not from the preparation to the implementation phase. This change was primarily associated with a greater use of the facilitator style to build capacity for independent action, provide direction, and suggest options versus asking questions or providing support to the teams. We also found that the coaches’ increased their use of the expert style during the sustainment phase. This may have been associated with the coach sharing their knowledge about sustainment, a concept often overlooked during the implementation of organizational change. The relationship between the use of specific coaching styles and implementation efficiency was not explored in this research.

Coaching should indeed be considered a “composite approach,” although a coach may utilize a particular style to a greater extent. While it is true that a given statement from the transcript is reflective of a discrete teaching style, the amalgam of statements that coaches make reflects their ability to adopt different coaching styles when responding to situational demands. As such, the coach did not solely rely on this singular coaching style despite the presence of one or more preferred coaching styles in each phase. Instead, their approach and style used when interacting with HSO staff varied during the calls as evident by the number of coded segments per interview. This finding is consistent with prior research which suggests that the coach engages in multiple activities when supporting efforts by the organization to implement change [8, 58].

Although these novel findings support the effectiveness of a qualitative approach to classify coaching styles, this study had several limitations. First, only 65% of all coaching calls had a recording that could be used to create a potential transcript for coding. Calls not recorded were typically due to technology issues or a coach forgetting to record the call. Consequently, the absence of available transcripts limited coding to 10 HSOs and represents an analysis based on only about half of the HSOs who received the ISF Strategy. Second, the inability to explore coaching styles across all participating HSOs limits the ability to draw any generalizable conclusions and about whether the pattern of use of a particular coaching style or combination of styles was associated with improved implementation effectiveness. Finally, it is possible that the purposive selection of interview transcripts for coding might have influenced the results, especially if a coach relied more on less-utilized coaching styles (e.g., delegator) with a HSO in one of the study phases (e.g., sustainment).

## Conclusion

Overall, this qualitative study indicates that it is feasible to use the Grasha-Riechmann framework as the conceptual foundation for coding transcripts from coaching calls within a facilitation-based implementation strategy. Unlike the Wizard of Oz, who strove to conceal his identity and “process” behind a curtain, we benefit by pulling the curtain aside to begin revealing what coaching styles are being utilized to implement a MIBI in HSOs. This glimpse provides insights into how coaches guide and teach staff throughout the implementation journey. Future investigations into coaching styles should employ a mixed-methods approach to compare the qualitative analyses of coaching transcripts to the fidelity of the ISF strategy and triangulate quantitative and qualitative data to better understand facilitation-based interventions and determine the influence of coaching styles on outcomes. With this knowledge, implementation researchers could tailor the coaching experience to prevalent coaching styles to improve the coaching experience. Specifically, coaching styles should be determined for the external facilitator or coach at the start of an implementation research study. If done in conjunction with a determination of the preferred learning styles of participants, the structure and content delivery mechanism for coach delivered content could be matched to the appropriate coaching style(s) to improve uptake by the study participants. A more tailored and structured approach might result in greater improvement and sustainment of implementation outcomes.

## Supplementary Information

**Additional File 1.** Description of Quality Improvement Collaborative Teaching Styles

**Additional File 2.** SAT2HIV Qual Codebook

**Additional file 3.** Standards for Reporting Qualitative Research (SRQR) checklist

**Additional file 4.** Overview of Coded Interview Segments

## Data Availability

The datasets analyzed during this study are not publicly available given privacy protections for individual level data collected for the study and the potential for re-identification of practice participants. Datasets are available from the corresponding author on reasonable request.

## Abbreviations

ATTC: Addiction Technology Transfer Center
HSO: HIV Service Organization
ISF: Implementation & Sustainment Facilitation
MI: Motivational interviewing
MIBI: Motivational interviewing-based intervention
QI: Quality improvement
QICTSI: Quality Improvement Coach Teaching Style Inventory
SAT2HIV: Substance abuse treatment to HIV
SUD: Substance use disorder
