# Supplementary material for: A peek behind the curtain: exploring coaching styles within the implementation and sustainment facilitation (ISF) strategy in the substance abuse treatment to HIV care study
Source: Implement Sci Commun. 2021 Dec 20;2:140. doi: 10.1186/s43058-021-00246-2 (PMC8686240; doi:10.1186/s43058-021-00246-2)
Supplement: Supplementary file 1 — Additional File 1. Description of Quality Improvement Collaborative Teaching Styles [file 43058_2021_246_MOESM1_ESM.docx]

**Additional File 1** Description of Quality Improvement Collaborative Teaching Styles

Delegator: Within this coaching style, you are concerned with developing the capacity in change leader and change team to function in an autonomous fashion. As a coach, you are working to cultivate self-directed, self-initiating learners within the organization. The goal is for change leader/change team will work independently on their quality improvement projects. While you are available at the request of change leader/change team as a consultant and resource person, your primary objective when using this coaching style is to give the change leader/change team in the organization the discretion to take independent action by providing a mechanism or tool that will enable to change team to immediately work independently.

Expert: In this coaching style, you often possess knowledge and expertise that the change leader needs to be successful. As a result, you may strive to maintain your status as a quality improvement expert by displaying detailed knowledge and by challenging change leader/change team to enhance their skills and competence with quality improvement tools and techniques. This knowledge may be based on your role as the coach in a quality improvement initiative. You are concerned with transmitting information and ensuring that the change leader/team are well prepared to implement change within their organization.

Facilitator: The goal of this coaching style is to develop in the change leader/team the capacity for independent action, initiative, and responsibility by emphasizing the personal nature of coach-change leader/team interactions. As a coach, you ask good questions designed to stimulate creativity and to encourage the change leader/team to explore options and make informed choices about change in their organization. In your coaching, you work with change leader/team on projects in a consultative fashion, suggesting alternative solutions, and try to provide as much direction, support, and encouragement as possible. When using a facilitator coaching style, you may also realize that you are offering encouragement, being empathetic, or reenforcing good ideas from the change leader/change team.

Formal authority: This coaching style places emphasis on the status you possess with the change leader/team that you are working with based on your knowledge and role as a quality improvement coach. When coaching, you are concerned with providing positive and negative feedback; establishing learning goals; and in ensuring that the change leader and change team are prepared to implement change in the organization. You may also feel the need to set expectations and rules of conduct for change leader/change team. When using this coaching style, you want to make sure that you provide information about the correct, acceptable, and standard ways to use quality improvement tools and techniques and to provide change leader/team with the structure they need to learn.

Personal model: In this coaching style, you believe in "teaching by personal example" and establish a prototype for how you like to think and behave when interacting with change leader/change team. When coaching, you prefer to oversee, guide, and direct the change leader/change team by showing them how to use a quality improvement tool (e.g., flowcharting) and encourage them to observe and then to emulate your approach. In the personal model of coaching, you may find that you are using phrases such as “*this is what I would recommend, or this is what I would do*” and you may tend to offer suggestions more frequently to the change team rather than let them explore options and make informed choices about change in their organization.
