# Supplementary material for: A peek behind the curtain: exploring coaching styles within the implementation and sustainment facilitation (ISF) strategy in the substance abuse treatment to HIV care study
Source: Implement Sci Commun. 2021 Dec 20;2:140. doi: 10.1186/s43058-021-00246-2 (PMC8686240; doi:10.1186/s43058-021-00246-2)
Supplement: Supplementary file 2 — Additional File 2. SAT2HIV Qual Codebook [file 43058_2021_246_MOESM2_ESM.docx]

**Additional File 2** SAT2HIV Qual Codebook

**Delegator:** You are concerned with developing team capacity to function in an autonomous fashion. Specific encouragement. Group is already working independently.

*Delegator Sub-themes*

1. **Being accessible to the team**: when a coach has given the team a task and the coach offers to help about how to carry it out.
2. **Providing specific directions:** More specific than “providing directions” in the facilitator coaching style.
3. **Being directive about expectations:** Coach was being directive and helping the team understand what was expected
4. **Between meeting expectations.** Giving them a specific expectations about what to do in between the meetings.

“So having a nice plan, as you already have, based on your description and your thinking down the road, in terms of months from now, how you'll be doing, say, six months from now, in terms of being able to recruit the numbers.”

1. **Affirming independent actions**: Giving the team the discretion to act in an effort to cultivate self-directed, self-initiating learners.

“You've been doing some marketing and letting people know that you're doing this. You have people who you're all set to go ahead and recruit and do the part one screen on and then randomize and do the part two. So you've got yourself in good position to get started.”

“But for the time being, what you're starting to systematize here is at least you're, you know, taking on this role of screening. And that's why, you know, as you were talking about what it will look like in Burrell in a couple of months as the study goes away, and, Bonnie, you were starting to think about how it would just look regularly that everybody's annual screening has this piece to it where they just regularly get that, that's very akin to that whole idea. And you just become this more end-of-the-branch sensor to tell the whole system, you know, what's needed.”

**Expert**: The coach possesses knowledge and expertise that the team needs to be successful. (even if there is a mention of a QI tool process – if its use or application is not described specifically it is still Expert). Coach is displaying detailed knowledge/info that the coach is aware of based on their role on the study and challenging the staff to enhance skills and competence with QI tools and techniques

“From my organizational change engineering perspective here, a couple things really stand out, and one is that, you know, you found it very beneficial to your environment, being able to get in and use that motivational interviewing technique to work better with the clients, to really get a, maybe a richer or deeper understanding of some of who they were. It seemed to be really important on that. So that seems to be a really great positive of doing that. “

General knowledge but not knowledge related to the project “I want to point out the CDC just ten days ago released some new data, released a new report of the newest data that they have, which is from 2011 to 2013, that the numbers, are you guys seeing a chart in front of you now?”

“And so, and Missouri has a little bit of work to do compared to the others. There’s really only two others in this list that are, that had the kind of performance that was less than 35.5%, or three. There’s West Virginia at about 25%. There was Illinois at 26%, District of Columbia just a half percent lower performance there with about 35% of HIV-infected folks being enrolled in regular care services. And so that, as I said, that report just came out ten days ago with that data.”

“And I, my quick read on that is that it was definitely Ryan White and more”

Facilitator: The personal nature of team interactions. General encouragement.

- Guide and direct by encouraging cooperative and independent learning activities, suggesting options
- Good at questions, exploring options, suggesting alternatives, and encouraging making informed choices
- Work on projects in a consultative fashion and provide direction, support, and encouragement
- Consultive – general

*Facilitator Sub-themes*

1. **Asking questions:** Good at questions, exploring options, , and encouraging making informed choices

“And is Nutmeg the IT vendor for you guys?” “Anything in terms of the research procedures? We're working through that business of uploading. Anything else in terms of, okay, what are we supposed to do and when are we supposed to do it and what about those brief screens and what about, you know, this business of recruiting people and randomizing people and all that? Any questions or confusions or concerns about any of that stuff?”

1. **Suggesting options** with the ultimate objective of promoting independent action.
2. **Capacity for independent action** (more distal than Delegator): Encouraging the team to develop their capacity for independent action, initiative and responsibility.

“it's just a matter of getting everything uploaded as soon as you have the technology figured out so that you can do that”

“So what, I mean, it’s kind of up to you, to Burrell now, at this point, to be able to screen clients. And so you could take the study screener and modify that for, you know, Burrell’s purpose.”

1. **Supportive action or provides support:**  the Coach offers to provide support such as connecting with other study team members.

“So let me try and see what we can do to move this along and/or to look for maybe an alternative workaround that hasn't been suggested yet.” “I will do that after we're done with our call today.”

1. **Providing direction:**

**“**The notion is that you could, if you're going to recruit through another provider, you might put it on their bulletin board or something like that.” “And then you can just plug in your own info in the appropriate spots on that”.

Exclude: questions related to setting agenda, setting meetings, but **include** questions related to participants’ willingness/interest in attending a meeting.

**Formal authority:** Providing information about tools and process, QI or field related. Also the status you possess with the team that you are working with based on your knowledge and role as QI coach.

*Formal Authority Sub-themes*

1. **Setting expectations and ensuring preparation:** Establishes learning goals, expectations, and rules of conduct

“[Study staff] will then start offering group coaching calls once a month. And you'll get these feedback reports on every single case that you do. These will be done by 1 of about 15 trained raters.”

1. **Providing Information:** Provides information about specific correct, acceptable, and standard ways to use QI tools and technique

“So if you're looking at the screen, you'll see that I flipped over to this other sheet. One of the things that we're going to be doing each month, in addition to keeping notes, as I have been, about the issues, and I should write down that we did some site visit planning, obviously. We are going to be using these three simple scales on these three issues because one of the things we want to keep track of is how we are doing in terms of recruiting the 12 participants each month, and then they get randomized, and implementing the 6 brief interventions each month, and then implementing at the highest-quality level possible. And then we have three drop-downs. We have drop-down scales for each of these. In terms of, we haven't started yet, so these are a little bit artificial, but I am going to ask you anyway to kind of give me a sense of how you think you're prepared or set in terms of being ready to do the 12 recruitments per month.”

“They all have been, you know, trained to rate roughly the same, and so you can kind of get this feedback report that you guys have received and help you kind of just, it's not supposed to be a score card. It's really supposed to be just sort of like a mechanism or a tool through which you, you know, show you areas that you're really strong on and areas that you might want to try to improve on.”

1. **Explaining learning structure or process:** Explaining learning structure/process about QI – specific application to the site

**“**You start to learn things like what kind of training works best? Did we do enough training? Is it more than enough training? For example, if we find out that everybody implemented absolutely perfectly, then you might go back to the drawing board and say, well, gee, maybe we overdid it. Maybe we could get along with half as much training and still get an excellent result. So it's part of how you understand how this training works and how it gets implemented. So those are part of the learnings that we'll get from the process.

“Because sometimes it's just administrative, people with administrative systems' privileges just have to give you access to particular sites. It sounds like it's more complicated than that.”

“here is how others are doing and how you could do it” (vs. expert “here is how others are doing it”

1. **Feedback**/**affirmation**: Provides positive and negative feedback,

“Good question, good question.” “So that's smart and wise.” “You're doing a good job of working through the pieces” “So that sounds good, so a little bit of communications on your end.”

“That makes sense. So, yeah, I mean, that's a natural sequence that the initial volume seems okay, and then, over time, you might start to be working through the clientele that you're actively serving and then saying, well, we could do some outreach.”

**“**That sounds reasonable, you know, given the status.”

“Good. Excellent. And no need to apologize because that's exactly what we're supposed to be doing. That's what this call is about, kind of working through the nuts and bolts in terms of getting ready to do this. So if this is the opportunity to chat, then perfect, that's the vehicle it's supposed to be.”

“You know, what I’m hearing in there is that even, well, I guess it wasn’t explicit in what you were saying because you didn’t mention that it takes up your time to do the brief intervention, but what I did hear a fair bit of is that there’s sort of an unassailable opportunity or a need to from, of these clients for this sort of brief intervention, for this attention on substance use, that its impact in the continuum there is certain and multiplicative, and that if there’s a chance to deliver some sort of assistance in this, that it’s needed. “

So, yeah, I mean, that's a natural sequence that the initial volume seems okay, and then, over time, you might start to be working through the clientele that you're actively serving and then saying, well, we could do some outreach.

**Personal Model**: You believe in "teaching by personal example" and establish a prototype for how you like to think and behave when interacting with team.

*Personal Model Sub-themes*

1. **Prior concrete experiences :** Encourage observing and emulating your approach through the specific use of suggestions or the use of phrases such as “what I would recommend” or providing explicit statements of how he would do it or has done it in the past (i.e., “what I would do”)**:**

“Well, if you are talking with [study team member] about it, that would be what I would be recommending. I would be connecting it to one of the research team staff members to work out the details.”

“That would be my first thought, that that could be an option, just to scan them and send them that way.”

One suggestion I would have with the recording, if you're finding that a lot of people are very anxious and get concerned about the recording, I mean, obviously, you're getting permission and, obviously, they need to know that you're recording them, so the recording has to be clear.  I mean, it's not like we're sneaking it in in our pocket like we're doing some investigation or something.  But when you introduce it and, you know, you've gotten permission to do it, try to have it be as casual as you can.

1. **Providing direction without being overly directive:** Coach teaches by example and prefers to oversee, guide, and direct by showing how to use a QI tool (e.g., flowcharting)

So if you're looking at the screen, you'll see that I flipped over to this other sheet. One of the things that we're going to be doing each month, in addition to keeping notes, as I have been, about the issues, and I should write down that we did some site visit planning, obviously. We are going to be using these three simple scales on these three issues because one of the things we want to keep track of is how we are doing in terms of recruiting the 12 participants each month, and then they get randomized, and implementing the 6 brief interventions each month, and then implementing at the highest-quality level possible.
