# Supplementary material for: A peek behind the curtain: exploring coaching styles within the implementation and sustainment facilitation (ISF) strategy in the substance abuse treatment to HIV care study
Source: Implement Sci Commun. 2021 Dec 20;2:140. doi: 10.1186/s43058-021-00246-2 (PMC8686240; doi:10.1186/s43058-021-00246-2)
Supplement: Supplementary file 4 — Additional file 4. Overview of Coded Interview Segments [file 43058_2021_246_MOESM4_ESM.docx]

**Additional file 4** Overview of Coded Interview Segments

|  | Number of Coded Segments | | |  |  | % of Coded Segments across Study Phase | | |
| --- | --- | --- | --- | --- | --- | --- | --- | --- |
| Coaching Styles | Preparation | Implementation | Sustainment | Total |  | Preparation | Implementation | Sustainment |
| **Delegator** | **61** | **60** | **29** | **150** |  | **40.7%** | **40.0%** | **19.3%** |
| **Expert** | **320** | **356** | **243** | **919** |  | **34.8%** | **38.7%** | **26.4%** |
| **Facilitator** | **481** | **587** | **369** | **1437** |  | **33.5%** | **40.8%** | **25.7%** |
| Facilitator-Asking questions | 320 | 399 | 220 | 939 |  | *34.1%* | *42.5%* | *23.4%* |
| Facilitator-Capacity for independent action | 33 | 60 | 52 | 145 |  | *22.8%* | *41.4%* | *35.9%* |
| Facilitator-Providing direction | 8 | 16 | 22 | 46 |  | *17.4%* | *34.8%* | *47.8%* |
| Facilitator-Suggesting options | 20 | 31 | 27 | 78 |  | *25.6%* | *39.7%* | *34.6%* |
| Facilitator-Supportive action or provides support | 100 | 81 | 48 | 229 |  | *43.7%* | *35.4%* | *21.0%* |
| **Formal Authority** | **786** | **938** | **431** | **2155** |  | **36.5%** | **43.5%** | **20.0%** |
| Formal Authority-Affirmation | 233 | 316 | 195 | 744 |  | *31.3%* | *42.5%* | *26.2%* |
| Formal Authority-Explaining learning structure or process | 61 | 47 | 27 | 135 |  | *45.2%* | *34.8%* | *20.0%* |
| Formal Authority-Feedback | 138 | 209 | 110 | 457 |  | *30.2%* | *45.7%* | *24.1%* |
| Formal Authority-Provide Information | 107 | 201 | 56 | 364 |  | *29.4%* | *55.2%* | *15.4%* |
| Formal Authority-Setting expectations or ensuring preparation | 254 | 194 | 58 | 506 |  | *50.2%* | *38.3%* | *11.5%* |
| **Personal Model** | **21** | **32** | **12** | **65** |  | **32.3%** | **49.2%** | **18.5%** |
| Total Coded Segments | 1669 | 1973 | 1084 | 4726 |  |  |  |  |
